# An update on the pathogenesis and diagnosis of Diamond–Blackfan anemia

**DOI:** 10.12688/f1000research.15542.1

**Published:** 2018-08-29

**Authors:** Lydie Da Costa, Anupama Narla, Narla Mohandas

**Affiliations:** 1 Université Paris 7 Denis Diderot-Sorbonne, Paris, France; 2 AP-HP, Hematology laboratory, Robert Debré Hospital, Paris, France; 3 INSERM UMR1134, Paris, France; 4 Laboratory of Excellence for Red Cell, LABEX GR-Ex, Paris, France; 5 Stanford University School of Medicine, Stanford, USA; 6 New York Blood Center, New York, USA

**Keywords:** Diamond-Blackfan anemia, Ribosomapthy, erythropoiesis, inherited bone marrow failure

## Abstract

Diamond–Blackfan anemia (DBA) is a rare congenital hypoplastic anemia characterized by a block in erythropoiesis at the progenitor stage, although the exact stage at which this occurs remains to be fully defined. DBA presents primarily during infancy with macrocytic anemia and reticulocytopenia with 50% of cases associated with a variety of congenital malformations. DBA is most frequently due to a sporadic mutation (55%) in genes encoding several different ribosomal proteins, although there are many cases where there is a family history of the disease with varying phenotypes. The erythroid tropism of the disease is still a matter of debate for a disease related to a defect in global ribosome biogenesis. Assessment of biological features in conjunction with genetic testing has increased the accuracy of the diagnosis of DBA. However, in certain cases, it continues to be difficult to firmly establish a diagnosis. This review will focus on the diagnosis of DBA along with a description of new advances in our understanding of the pathophysiology and treatment recommendations for DBA.

## Introduction

Diamond–Blackfan anemia (DBA) was described for the first time in the 1930’s as a constitutional hypoplastic anemia ^1, 2^. There was a gap of almost 60 years after the first description of the disease ^2, 3^ before the first gene was identified in DBA, namely ribosomal protein (RP) S19 ( *RPS19*) in 1999 ^4^. Surprisingly, for a disease in which the major defect is disordered erythropoiesis, the genes involved in the disease belong to both small and large subunits of the ribosome, which would be expected to have widespread consequences. The mutant RP is responsible for a defect in rRNA maturation, which is the signature feature for most DBA cases. DBA was indeed described as the first ribosomopathy in 2005 ^5^. Since this original classification, the actual definition of DBA is evolving with further developments in the genetic basis for the disorder, with the discovery of new DBA genes that are not directly involved in ribosome biogenesis. In this review, we will update these new insights into our understanding of DBA.

## Clinical features

DBA typically presents in infancy, most commonly with pallor and lethargy, at an estimated incidence of seven cases per million live births within some families who have a history of the disease. The median age at presentation is 8 weeks, with a median age at diagnosis of 12 weeks. There have been cases of hydrops fetalis ^6, 7^. The male-to-female ratio of cases is approximately 1:1 despite rare cases of X-linked inheritance. More than 90% of the reported cases present clinically by 1 year of age. DBA is characterized by a macrocytic moderate or severe anemia in association with aregenerative bone marrow and reticulocytopenia. The disorder is also characterized by elevated erythrocyte adenosine deaminase (eADA) activity in over 75% of cases.

The DBA Registry of North America (DBAR), a database of more than 700 patients, was established in 1991 and provides important information regarding the epidemiology and biology of DBA ^8– 12^. Almost half of patients exhibit physical abnormalities, except short stature, which is a known feature of DBA but could also be a result of chronic anemia, iron overload, corticosteroid administration, or a combination of all three. Included in the constellation of physical anomalies are a high number of craniofacial anomalies (50% of patients), upper limb and hand––in particular thumb (38%)––abnormalities, and genitourinary (39%) as well as cardiac (30%) abnormalities.

DBA is recognized as a cancer predisposition syndrome with an observed to expected ratio for all cancers of 5.4. The most common malignancies were MDS, AML, colon carcinoma, osteosarcoma, and genitourinary cancers ^12– 15^.

Of interest, there appears to be no genotype–phenotype correlation with regard to steroid responsiveness, remission, or cancer predisposition. However, *RPL5* gene mutations have been associated with cleft palate malformation and are the most important rate of malformations in DBA cases ^16, 17^, while *RPL11* mutations are associated with the classic triphalangeal thumb ^17^. Recently, mutations in the *RPL15* gene have been identified in cases of hydrops fetalis in DBA patients ^7^, and *RPL35a* gene mutations are associated with neutropenia.

## Biological features

DBA is one of the inherited bone marrow failure (IBMF) syndromes that include Fanconi anemia, Shwachman–Bodian–Diamond syndrome, dyskeratosis congenita, and cartilage hair hypoplasia ^18– 24^. All of these syndromes have a quantitative defect in hematopoiesis. Among the IBMF syndromes, DBA is unique in that it involves a specific intrinsic quantitative defect in erythropoiesis ^25^.

There is strong evidence that the erythroid blockage likely occurs between the BFU-e and CFU-e stage of erythroid development ^26^. It should be noted that some previous reports have suggested a general blockade upstream during hematopoiesis, since long-term culture experiments have shown a defect in megakaryocytic and granulocytic progenitors ^27, 28^ and there are rare cases of DBA which progress to a complete aplasia ^14, 29^.

The erythroid blockade is responsible for the erythroblastopenia characterized by the absence or less than 5% of erythroid progenitors in the bone marrow aspirate or an important paucity of the erythroid progenitors in the bone marrow biopsy in an otherwise normal bone marrow with no qualitative dyserythropoiesis or defects in other hematopoietic cell lineages. Neutropenia and thrombocytopenia, and in some instances thrombocytosis, have been described at diagnosis or during DBA evolution, implying that DBA diagnosis should not be ruled out when these particular blood cell anomalies are noted at DBA presentation.

Strikingly, DBA is associated with an increased eADA activity ^30– 33^. eADA is a critical enzyme of the purine salvage pathway, which enables the deamination of adenosine in inosine and 2'-deoxyadenosine deamination in deoxyinosine. In the French registry of over 300 DBA patients, eADA has been found to be elevated in 90% of non-transfused DBA patients as reported in a previous study ^32^ and in 75% of DBA patients from the American registry with a sensitivity of 84%, specificity of 95%, and positive and negative predictive values of 91% for the diagnosis of DBA compared with other IBMF syndromes ^31^. While an elevated eADA activity is a strong feature of DBA, it is also increased in some leukemias, lymphomas, and immune system disorders ^34^. The challenge in performing eADA testing is that the test is not routinely available and is currently performed in only one lab in each of the following countries: the USA, France, Germany, Italy, Poland, Israel, and Turkey ^35^. It should be noted that the test needs to be performed on fresh blood samples or samples stored at 4°C for less than a few days and on samples prior to red cell transfusions.

In order to eliminate the most frequent differential diagnosis, namely a parvovirus B19 infection, parvovirus B19 serology (IgM/IgG) or parvovirus B19 PCR in the blood (or in the bone marrow, which has a higher sensitivity) is mandatory.

The other biological tests that may be useful in DBA diagnosis are 1) the erythropoietin (EPO) level, which is consistently elevated in DBA as a result of a lack of effective erythropoiesis with a normal kidney response to the anemia and a quantitative deficiency of the EPO receptors that bind EPO due to the large decreases in the number of erythroid precursors, and 2) immunophenotyping and IgG/IgA agglutinin titer. A DAT test in association with an erythroid clonogenic *in vitro* culture assay with and without the patient’s sera may be helpful in rare cases, mostly in adults, in order to eliminate immune erythroblastopenia in doubtful DBA cases.

## Molecular diagnosis

**Figure 1.**
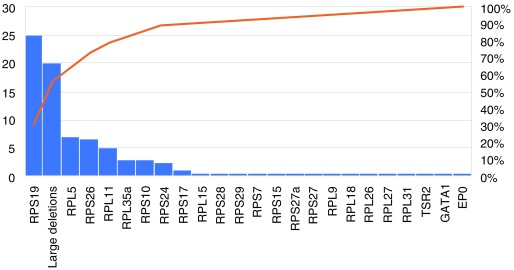
Representation of the frequency of the mutated genes involved in Diamond–Blackfan anemia (DBA) from DBA-affected populations all over the world (literature data).

The first gene associated with DBA was identified in 1999 in a Swedish patient with DBA ^4, 36^ who carried a balanced translocation between the chromosomes X and 19. Since DBA exhibits a 1:1 sex ratio, it was thought unlikely to be of X-linked inheritance, and the candidate gene in 19q chromosomal breakpoint was explored and identified. Surprisingly, for a disease with an erythroid tropism, the identified gene was an RP from the small ribosome subunit gene *RPS19*. Subsequently, mutations or deletions in 19 other RP genes have been identified by whole exome/genome sequencing and CGH/SNP array. These include *RPL5, RPL11, RPL35a, RPS10, RPS24, RPS17, RPL15, RPS28, RPS29, RPS7, RPS15, RPS27a, RPS27, RPL9, RPL18, RPL26, RPL27,* and *RPL31* as well as three other non-RP genes, *TSR2*, *GATA1*, and *EPO* ( Table 1, Figure 1, and ^4, 16, 17, 35, 37– 49^). It is still debated if the disease associated with non-RP genes is classical DBA or “DBA-like” disease.

**Table 1.** Genes involved in Diamond–Blackfan anemia (DBA) from the most mutated to reported cases, incidence, and references.

Mutated gene	Incidence in DBA population	References
*RPS19*	25%	Draptchinskaia *et al.* ^4^ Willig *et al.* ^51^ Ramenghi *et al.* ^55^ Cmejla *et al.* ^56^ Proust *et al.* ^57^ Campagnoli *et al.* ^58^
Large deletions	10–20%	Gustavsson *et al.* ^59^ Quarello *et al.* ^60^ Farrar *et al.* ^39^ Quarello *et al.* ^49^ Kuramitsu *et al.* ^48^
*RPL5*	7%	Gazda *et al.* ^17^ Cmejla *et al.* ^16^ Quarello *et al.* ^61^
*RPS26*	6.6%	Doherty *et al.* ^37^
*RPL11*	5%	Gazda *et al.* ^17^ Cmejla *et al.* ^16^ Quarello *et al.* ^61^
*RPL35a*	3%	Farrar *et al.* ^38^
*RPS10*	3%	Doherty *et al.* ^37^
*RPS24*	2.4%	Gazda *et al.* ^41^
*RPS17*	1%	Cmejla *et al.* ^42^ Song *et al.* ^62^
*RPL15*	One case Six cases	Landowski *et al.* ^40^ Wlodarski *et al.* ^7^
*RPS28*	Two families	Gripp *et al.* ^52^
*RPS29*	Two families	Mirabello *et al.* ^45^
*RPS7*	One case	Gazda *et al.* ^17^
*RPS15*	One case	Gazda *et al.* ^17^
*RPS27a*	One case	Gazda *et al.* ^17^
*RPS27*	One case	Wang *et al.* ^63^
*RPL9*	One case Two cases	Gazda *et al.* ^17^ euroDBA group, in preparation
*RPL18*	One family	Mirabello *et al.* ^64^
*RPL26*	One case	Gazda *et al.* ^65^
*RPL27*	One case	Wang *et al.* ^63^
*RPL31*	One case	Farrar *et al.* ^66^
*TSR2* (X-linked)	One family	Gripp *et al.* ^52^
*GATA1* (X-linked)	Five families	Sankaran *et al.* ^43^ Klar *et al.* ^67^ Ludwig *et al.* ^53^ Parrella *et al.* ^68^
*EPO*	One case	Kim *et al.* ^44^

DBA is thus a polygenic disease with mutations in 20 of the 80 RP genes that code for the complete ribosome. Interestingly, mutations including deletions in six of 20 identified genes, namely *RPS19, RPL5, RPS26, RPL11, RPL35a*, and *RPS24*, account for 70% of all DBA cases ( Figure 1). Some RP gene mutations exhibit a phenotype–genotype relationship (see “clinical features” above). All the RP mutations identified to date are heterozygous; homozygosity is thought to be lethal, which has been confirmed in zebrafish and murine models of DBA ^50^. Multiple pathogenic RP mutations have not been reported in any DBA patient to date. Except for *RPS19* ^51^, no hot spot regions in the DBA genes have been reported. Various types of mutations may be associated with DBA phenotype, although most *RPS19* gene mutations are missense mutations, while nonsense mutations are more frequent in *RPL5*- and *RPL11*-associated DBA. Large deletions have been reported in 20% of patients, which makes large deletions the second most frequent genetic defect after *RPS19* gene mutation (25%) in DBA.

With regard to the “non-RP” genes linked to DBA, *TSR2* is known to play a role in ribosome biogenesis, since it is involved in the pre-rRNA processing and binds to RPS26 ^52^. GATA1 is the major erythroid transcription factor and plays a critical role in regulating normal erythroid differentiation by activating an array of erythroid genes. Ludwig *et al.* ^53^ have shown that GATA1 transcripts are specifically less translated compared to others in DBA owing to a higher threshold for initiation of translation of GATA1 mRNA due to defective ribosomal biogenesis. Gastou *et al.* ^54^ showed that HSP70, the chaperon of GATA1, is degraded by the proteasome following polyubiquitination during the BFU-E and CFU-E stages of erythropoiesis. Decreased HSP70 expression has been noted in all of the RP mutated-gene-tested DBA patients and in shRNA models other than RPS19, which exhibit a normal expression of HSP70. This correlates perfectly with the low level of induced apoptosis in these RPS19-mutated DBA patients compared to the RPL5- or RPL11-mutated ones. Interestingly, HSP70 degradation is responsible for caspase-3-dependent GATA1 cleavage during terminal erythroid differentiation and the resultant decreased GATA1 protein expression at late stages of erythroid differentiation ^54^. Finally, the *EPO* gene has been found to be mutated in one consanguineous Turkish family in the USA, with a homozygous missense mutation in exon 5 of *EPO*. The pathogenicity of this mutation has been validated *in vitro* by functional analysis with a well-established defect in erythroid proliferation and differentiation ^44^.

Following detailed mutational screening analysis using various methods, no molecular defect can be documented in 20 to 30% of DBA cases. It is likely that mutations in a regulatory region including intronic regions and promoters in one of the known RP genes may account for the DBA phenotype. It is also important to emphasize that the first step in molecular diagnosis should always be to characterize the phenotype including family history, pregnancy complications, congenital malformations, and characteristics of the anemia including an evaluation of the bone marrow, which we still believe is an essential part of the evaluation. Indeed, hypoplastic anemia can be seen as a part of some congenital dyserythropoietic anemias (CDAs) ^69^ and acquired minus 5q syndrome ^70^; therefore, the frontier between DBA and these syndromes may sometimes be difficult to ascertain without a meticulous clinical and microscopic examination by an expert hematologist and hematopathologist.

## DBA pathophysiology

In terms of the history of insight into DBA as a disease, there was widespread use of erythroid cell cultures from the 1950’s to the end of the 1990’s prior to the advent of the molecular biological approaches. In the earlier stages, immune mechanisms (humoral or cell-mediated immune inhibition by T-cytotoxic or T-helper lymphocytes) were thought to play a role in the pathophysiology of DBA owing to the effectiveness of corticosteroid therapy in correcting the anemia phenotype ^71– 73^. However, no conclusive evidence in support of immune-meditated suppression of erythropoiesis could be documented.

A defective microenvironment as a major cause of DBA pathophysiology was also ruled out because of the effectiveness of bone marrow transplantation as a curative treatment in DBA and also from findings that documented normal erythroid proliferation of control CD34 ^+^ cells cultured in a DBA microenvironment ^28, 74^. DBA can thus be defined as an intrinsic defect in erythropoiesis due to defective ribosome processing. However, the exact stage at which blockade occurs during erythropoiesis still needs to be fully defined. The best documented study ^26^ implies a blockade between the BFU-E and the CFU-E stages or between the EPO-independent and the EPO-dependent stages of erythroid development. However, data from erythroid culture studies from DBA patients are highly heterogeneous and depend on a number of variables including the erythroid culture methods used. There is, however, some convincing evidence that different RP mutations exhibit significant differences in the erythroid proliferation phenotype ^54, 75^.

The major unresolved questions in DBA remain how a defect in RP is responsible for a specific defect in erythropoiesis and why there is a different penetrance of the same mutation among different individuals. Red blood cells do exhibit a high level of cell production (2 × 10 ^11^/day) in humans, and the level of protein translation needed is indeed very high, which may explain, in part, the erythroid tropism of DBA. In this context, any factor that affects GATA1 expression would be deleterious in DBA as well. Indeed, HSP70 ^54^, and the recently reported ribonuclease inhibitor 1 (RNH1) ^76^ that binds to the 40S ribosome small subunit, could be involved in the translational control of GATA1 and consequently affect erythropoiesis, resulting in the DBA phenotype. In addition, ribosome amounts have been shown to be critical for the cell lineage commitment. Recently, a global reduction in ribosome levels in DBA has been documented, while the ribosome composition was normal, which altered the translation of specific RNA transcripts ^77^. These exciting new discoveries are enabling the establishment of a link among a mutated RP gene, a defect in ribosome biogenesis, and the specific effect on erythropoiesis in DBA.

p53 also plays an important role in DBA pathophysiology, with p53 activation and the altered expression of its targets (p21, Bax, noxa) having been documented in erythroid cells from affected patients and in CD34 ^+^ cord blood erythroid progenitors following lentiviral transfection with various shRNAs targeting RPS19, RPL5, or RPL11 transcripts ^75, 78^. The relationship between p53 and GATA1 is well established, with GATA1 inhibiting p53 ^79^. It has also been shown that wild-type HSP70 overexpression, by restoring GATA1, was also able to decrease p53 activation (phosphorylated p53) in RPL5- and RPL11-depleted erythroid cells ^54^. Decreased expression of GATA1 may thus account for p53 activation in DBA. Indeed, activation of p53 in DBA may be due to not only the nucleolar stress and overexpression of some RPs (RPS3, RPS7, RPS27, RPS27a, RPL5, RPL11, and RPL23), which are able to directly bind MDM2 in order to release the p53/MDM2 binding, but also the defect in GATA1 in DBA.

Another level of complexity in DBA involves the role of free excess heme in DBA pathophysiology due to imbalance between decreased globin synthesis and excess of free heme, which can generate reactive oxygen species and increased apoptosis, leading to the death of erythroid progenitors and precursors. Autophagy and cell metabolism have also been shown to be important in DBA pathophysiology ^80, 81^.

Thus, it appears that deciphering the complex interplay between the multiple mechanisms identified to date, and perhaps others yet to be discovered, is needed to develop detailed understanding of the documented variations in clinical severity of the highly heterogeneous DBA phenotype.

## Therapeutic options

The current standard of care for DBA includes corticosteroids and/or chronic transfusions with the only definitive treatment (for the hematologic complications) being bone marrow transplantation. Approximately 80% of patients respond initially to corticosteroids with an improvement in, or complete remission of, their anemia. However, prolonged corticosteroid treatment has been problematic for many patients such that only about 40% will remain on corticosteroids for a considerable period of time. With existing treatments, the overall survival of patients, as reported by the DBAR, is 75% at 40 years of age; median overall survival is 58 years. As our understanding of the pathophysiology of the ribosomopathies increases, the goal is to be able to translate these findings into novel therapeutic options for patients with DBA.

Recently, the use of hematopoietic stem cell transplantation (HSCT) in DBA patients is increasing and has provided encouraging results. August and colleagues reported the first successful transplant for DBA in 1976 ^82^. Alter reviewed stem cell transplantation in DBA in 1998 ^83^; in an analysis of 35 of the 37 cases reported until that point, the actuarial survival for primarily allogeneic HLA-matched donor transplants was 66%. Recent work has proposed that this figure is approximately 90% for matched, related HSCT in young, otherwise healthy patients ^84, 85^. The latest analysis from the DBAR found that HLA-matched related-donor transplant resulted in an overall survival of 76.9 ± 8.4% and, for patients aged 9 years or younger, survival was 93.8 ± 6.1%. In transplants from an unrelated donor, the overall survival showed an improvement: in 1994 to 1999, it was reported to be 32.1 ± 11.7%, and in 2000 to the present, it was 85.7 ± 13.2% ^11^. A “How I Treat” article by Vlachos and Muir ^86^ provides a detailed approach to the treatment of DBA. There are also several open clinical trials (details available at clinicaltrials.gov) for patients with DBA and more in development.

## Conclusion

DBA, a rare congenital hypoplastic anemia characterized by a block in erythropoiesis, is most frequently due to a sporadic mutation in genes encoding several different RPs. DBA was indeed the first identified human ribosomopathy ^5^. The erythroid tropism of the disease is still a matter of debate for a disease related to a defect in global ribosome biogenesis. Assessment of biological features in conjunction with genetic testing have increased the accuracy for the diagnosis of DBA. The current standard of care for DBA includes corticosteroids and/or chronic transfusions, with the only definitive treatment being bone marrow transplantation.
